# The cost of hemiarthroplasty compared to that of internal fixation for femoral neck fractures: 2-year results involving 222 patients based on a randomized controlled trial

**DOI:** 10.3109/17453674.2010.492763

**Published:** 2010-07-16

**Authors:** Frede Frihagen, Gudrun M Waaler, Jan Erik Madsen, Lars Nordsletten, Silje Aspaas, Eline Aas

**Affiliations:** ^1^Department of Orthopedics, Oslo University Hospital; ^2^Institute of Health Management and Health Economics, University of Oslo; ^3^Faculty of Medicine, University of OsloNorway

## Abstract

**Background and purpose:**

There is very little information on the cost of different treatments for femoral neck fractures. We assessed whether total hospital and societal costs of treatment of elderly patients with displaced femoral neck fractures differ between patients operated with internal fixation or hemiarthroplasty.

**Methods:**

222 patients (mean age 83 years, 165 women (74%)) who had been randomized to internal fixation or hemiarthroplasty were followed for 2 years. Resource use in hospital, rehabilitation, community-based care, and nursing home use were identified, quantified, evaluated, and analyzed.

**Results:**

The average cost per patient for the initial hospital stay was lower for patients in the internal fixation group than in the hemiarthroplasty group (€9,044 vs. €11,887, p < 0.01). When all hospital costs, i.e. rehabilitation, reoperations, and formal and informal contact with the hospital were included, the costs were similar (€21,709 for internal fixation vs. €19,976 for hemiarthroplasty). When all costs were included (hospital admissions, cost of nursing home, and community-based care), internal fixation was the most expensive treatment (€47,186 vs. €38,615 (p = 0.09)).

**Interpretation:**

The initial lower average cost per patient for internal fixation as treatment for a femoral neck fracture cannot be used as an argument in favor of this treatment, since the average cost per patient is more than outweighed by subsequent costs, mainly due to a higher reoperation rate after internal fixation.

## Introduction

The cost of hip fractures is high. For the individual suffering a hip fracture, there is a physical and a psychological cost. For society, there are costs for medical attention such as hospital treatment, rehabilitation, and an increased level of care. Intracapsular femoral neck fractures constitute approximately half of all hip fractures (Lofthus et al. 2001, Gjertsen et al. 2008). Most are displaced, and the main alternative treatments are internal fixation and arthroplasty. Meta-analyses have failed to demonstrate any clear difference in functional results (Lu-Yao et al. 1994, Bhandari et al. 2003, Parker and Gurusamy 2006, Rogmark and Johnell 2006), but the most recent studies have reported better hip function and higher health-related quality of life after arthroplasty (Rogmark et al. 2002, Blomfeldt et al. 2005, Keating et al. 2006, Frihagen et al. 2007). A high reoperation rate after internal fixation has been a consistent finding in both randomized and non-randomized studies; a Cochrane review found a revision rate of 36% for internal fixation and 11% for arthroplasty (Parker and Gurusamy 2006).

In the randomized trial that the present study is based on, reoperation rates of 42% for internal fixation and 10% for hemiarthroplasty were found (Frihagen et al. 2007). Morbidity and mortality rates are similar between the methods, even though arthroplasty surgery is more extensive with longer surgical time and more bleeding (Lu-Yao et al. 1994, Rogmark et al. 2002, Bhandari et al. 2003, Blomfeldt et al. 2005, Rogmark and Johnell 2006, Parker and Gurusamy 2006, Keating et al. 2006, Frihagen et al. 2007). We analyzed hospital and societal costs for hemiarthroplasty and internal fixation after displaced intracapsular femoral neck fractures, basing the data on a randomized controlled trial from Norway (Frihagen et al. 2007).

## Patients and methods

### Patients

Patients aged 60 years or older with an intracapsular femoral neck fracture with angular displacement in either radiographic plane (except the purely valgus impacted fractures with no displacement on the lateral view) who were previously ambulant were recruited for the study. Exclusion criteria were: being found unfit for arthroplasty surgery, having had previous symptomatic hip pathology, or having had a delay of more than 4 days from injury to treatment. Cognitive failure was not an exclusion criterion. Patients who were able to give informed consent did so. Patients who could not give informed consent because of temporary or permanent cognitive impairment were included after obtaining consent from the family.

222 patients were randomized to internal fixation (n = 112) or hemiarthroplasty (n = 110) between Sept 2002 and March 2004. Further information on patients, methods, and outcome has already been reported (Frihagen et al. 2007). The demographics of the patient groups were similar at baseline (Table 1).

**Table 1.** Baseline and demographic characteristics of patients with femoral neck fractures according to randomization group. Figures are numbers (percentages) unless otherwise stated

	Internal fixation (n = 112)	Hemiarthroplasty (n = 110)
Unable to give informed consent	24 (21)	27 (25)
Mean (SD) age at fracture	83 (7.7)	83 (7.3)
Women	87 (78)	78 (71)
ASA (American Society of Anesthesiologists) group I or II	59 (53)	51 (47)
Living situation before fracture base (n = 112 and 108) ^**a**^		
Home care	83 (74)	87 (80)
Nursing home	29 (26)	21 (19)
Mean (SD) retrospective Harris hip score (n = 109 and 100) ^**a**^	84 (15)	84 (14)
Previously recognized cognitive failure	40 (36)	29 (26)
Concurrent symptomatic medical disease	52 (46)	64 (58)
Concurrent condition or impairment likely to affect rehabilitation	74 (66)	73 (66)
Ability to walk without any aid (n = 112 and 107) ^**a**^	67 (60)	60 (56)
Fall from standing height or lower	109 (97)	109 (99)
Mean (SD) time from injury to admission (h) (n = 94 and 83 ^**a**^)	8.0 (14)	5.5 (15)

^**a**^ Data missing for some patients

### Interventions

Patients underwent either a closed reduction and internal fixation with 2 parallel cannulated screws (Olmed; DePuy/Johnson and Johnson, Sweden) or a Charnley-Hastings bipolar cemented hemiarthroplasty (DePuy/Johnson and Johnson, UK). The surgeons on call carried out all operations. The postoperative regimen was the same for the two groups, except that antibiotic prophylaxis was used in the hemiarthroplasty group. Early mobilization was encouraged, with weight bearing as tolerated. Both interventions had been standard operations in the department before the study (Frihagen et al. 2007).

### Costs

The study's time horizon was 2 years, with follow-up at 4, 12, and 24 months. By examining the patients' whereabouts and actual resource use, we obtained an estimate of cost per patient over time. Patient flow varied between the groups, e.g. patients who underwent primary hemiarthroplasty spent more time in hospital during the first stay, while patients who underwent internal fixation as primary operation had a higher incidence of reoperations (Figure). The costs were divided into 3 categories: (1) hospital costs as a direct consequence of the fracture, including both primary operations and reoperations, with rehabilitation and other contacts, i.e. out-patient consultations and telephone contacts; (2) hospital costs due to treatment(s) other than for the femoral neck fracture; and (3) costs due to changes in living situation (from independent living to nursing home) and the need for help in everyday life (home nursing or other home-based services and physiotherapy).

**Figure:**
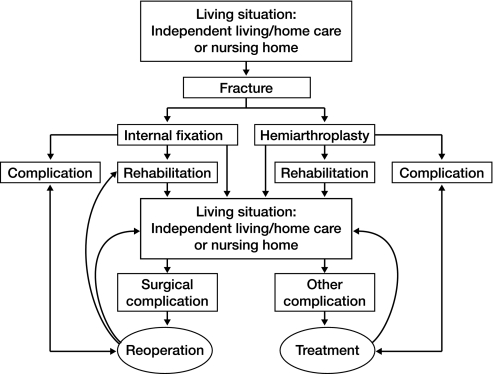
Flow chart of patients with hip fracture randomized to either internal fixation or hemiarthroplasty.

Use of most resources was registered prospectively during the study. Some costs, however, were identified from the hospital registry or based on expert opinion (Table 2). For some costs of the hospital stay, we used the average cost per unit from a cost–per-patient (CPP) analysis performed by the hospital administration. The CPP is arrived at by processing the cost per patient of each hospital contact, i.e. outpatient or inpatient, by means of the bottom-up method. CPP is either given as a fixed amount per patient per occurrence, or as a cost per hour consumed (Table 2). Cost of taxi and ambulance transportation was calculated from information from Norwegian Patient Transportation and from the hospital ambulance service. Costs of medication were taken from the Norwegian Hospital Pharmaceuticals. Laboratory costs and costs for outpatient consultations were taken from NAV (the Norwegian Labor and Welfare Administration). The total cost per operation was the sum of costs for equipment, including implants and other single-use equipment, anesthesia costs, and costs for the staff involved in the surgery. Postoperative costs were given as the real cost in the postoperative ward over a year divided by the number of patients in the department, controlling for different lengths of stay in hours. Telephone contact was valued as 15 min of a surgeon's time plus a minor capital cost. Rehabilitation was valued using diagnosis-related groups (DRG) both for hospital rehabilitation and private rehabilitation. DRG is a coding system used for administration of both clinical and financial activity in specialist healthcare. All DRG groups are given a weighting to reflect the intensity of treatment relative to the average patient. The cost for a DRG group is estimated by multiplying the cost weighting for that specific DRG group by the cost of one DRG. In 2006, the cost of one DRG was €3,513 (Norwegian Health Directorate, 2007). For patients who were in a nursing home for only a short period after the operation, the cost per week was calculated from the cost per year. The mean length of (rehabilitation) stay in a nursing home after hospital discharge was estimated to be 3 weeks, based on information from local providers.

**Table 2.** Identification, valuation and quantification of the healthcare resources. Costs are given in euros and have been adjusted to costs in 2006

Cost categories	Unit	Source of consumption data				Valuation	Unit price
		Hospital registry	Study registry		Expert opinion		Euros
			registered	interviews			
**1. Hospital and rehabilitation costs due to the femoral neck fracture**							
*Transportation*							
Ambulance	Rides			x		Average cost UUS ^**a**^	222
Taxi	Rides			x		Average cost UUS ^**a**^	44
Emergency ward	Visits			x		CPP ^**b**^	356
*Equipment (in surgery)*							
Internal fixation	Operations		x			Real costs	310
Hemiarthroplasty	Operations		x			Real costs	1,282
Removal of screws	Operations		x			Real costs	171
Soft tissue debridement	Operations		x			Real costs	946
Excision arthroplasty	Operations		x			Real costs	316
Open reduction	Operations		x			Real costs	709
Constrained acetabular cup (Avantage)	Operations		x			Real costs	1,238
Bipolar hemi-cup (Hastings)	Operations		x			Real costs	652
*Anesthesia costs*							
Fixed anesthesia costs	Operations		x			CPP ^**b**^	87
Variable anesthesia costs	Hours		x			CPP ^**b**^	63
*Personnel costs (in surgery)*							
Orthopedic surgeon, specialist	Hours		x			Wages	51
Orthopedic surgeon, in training	Hours		x			Wages	39
Anesthesiologist, in training	Hours		x			Wages	39
Nurse, surgery	Hours		x			Wages	27
Nurse, anesthesia	Hours		x			Wages	27
Post operative costs	Hours	x				Real costs	69
*Inpatient days*							
Overhead costs	Days		x			CPP ^**b**^	378
Direct attention	Days		x			CPP ^**b, c**^	201
Common costs	Days		x			CPP ^**b**^	250
*Medication/blood transfusion*							
Cefalotin (Keflin)	Dose			x		Real costs	6
Dalteparin, LMWH (Fragmin)	Dose			x		Real costs	1
Oxycodone (Oxycontin)	Dose			x		Real costs	1
Codein/paracetamol (Pinex Forte)	Dose			x		Real costs	0.2
Lactulose	Dose			x		Real costs	0.1
Paracetamol	Dose			x		Real costs	0.1
Blood	SAG		x			Real costs	143
*Radiology*							
Radiology images	X-rays				x	NAV ^**d**^	56
*Laboratory*							
All surgeries except soft tissue debridement	No				x	NAV ^**e**^	87
Soft tissue debridement	No				x	NAV ^**f**^	177
*Rehabilitation*							
Hospital rehabilitation	Days		x			DRG ^**g**^	632
Private rehabilitation	Days	x	x			DRG ^**g**^	632
Nursing home (short stay)	Days	x	x			KOSTRA ^**h**^	437
*Other contact*							
Out patient clinic	Visits			x		NAV ^**j**^	173
Telephone contact	Contacts			x		Wage	19
**2. Other hospital costs (other than treatment related to femoral neck fracture)**							
Type of treatment	DRG weight	x				DRG ^**k**^	Weight 3,513
**3. Costs due to changes in living situation**							
*Independent living/ home care*							
Practical assistance	Hours			x		Charge	41
Home nursing day	Hours			x		Charge	36
Home nursing night/weekend/public holiday	Hours			x		Charge	50
Administration	Months				x	Charge	67
Household costs	Year			x		KOSTRA ^**h**^	24,415
*Nursing home*							
Nursing home	Year			x		KOSTRA ^**h**^	75,881
Physiotherapy	Hours			x		Charge	25

^**a**^ UUS; Ulleval University Hospital

^**b**^ CPP: cost per patient (Ulleval University Hospital, 2006).

^**c**^ Patient categorization: categorizes the patients need for direct care on a scale that makes it possible to give a cost for different patient groups.

^**d**^ NAV: Norwegian Labor and Welfare Administration, primary category 104.

^**e**^ NAV: Norwegian Labor and Welfare Administration, codes 703a, 703c, 703d and 707a.

^**f**^ NAV: Norwegian Labor and Welfare Administration, codes 703a, 703c, 703d, 704a, 704b, 704c, 705a and 707a.

^**g**^ DRG 2006: secondary rehabilitation for DRG 210 weighted 0.18 (Norwegian Health Directorate 2009).

^**h**^ KOSTRA; Municipality-state-reporting in Statistics Norway.

^**j**^ NAV: Norwegian Labor and Welfare Administration, code A02, primary category 104 and out-of-pocket payment 31euros.

^**k**^ DRG 2006: 62 different DRG codes (minimum weight 0.26, maximum weight 4.5 and mean weight 1.36).

Other hospital costs were calculated using DRG weights from 2006. The patient's living situation and consumption of other municipal resources was registered at baseline and at the 4, 12, and 24-month follow-ups. Using KOSTRA (municipality-state registration) at Statistics Norway, we found that the mean running costs of living in a nursing home were €75,881 per year, while the average household costs for the patient group living at home (independent living and home care were assumed to be similar) was €24,415 (Municipality State Registration, 2009). Based on information from care providers in Oslo, home nursing care and practical assistance was estimated to be €36 per h during daytime and €50 at night, at weekends, and on public holidays with an added administration cost of €66 per month per patient. Physiotherapy was estimated to cost €25 per hour. The costs after a patient's death were cut immediately, as the relocation of resources is rapid. All costs in the study are given in euros (€1 = NOK9) and they were adjusted to 2006 values, with a discount rate of 4%.

### Statistics

Because of the high mean age and the fact that a proportion of the patients were very frail, the data set may have included extreme points. Cost data are often positively skewed; thus, the non-parametric bootstrap method was used. Bootstrapping is used to estimate a new standard error, variation, and mean by drawing a random sample with replacement and constructing a number of equally sized resamples of the existing dataset. We performed bootstrapping with 1,000 repetitions. Based on the bootstrap samples, a 2-sided independent sample t-test was used to test for differences in costs between the intervention groups. When testing for difference in variance in more than 2 groups, analysis of variance (ANOVA) was used. Statistical analysis was performed with SPSS 16.0 and STATA 10.

### Ethics

The study was conducted according to the Declaration of Helsinki and approved by the Regional Ethics Committee of Eastern Norway (June 18, 2002; 262-02103). The study was registered with ClinicalTrials.gov (NCT00464230).

## Results

### Comparison of cost of internal fixation and hemiarthroplasty

The accumulated costs of the first stay in hospital were €9,044 for internal fixation and €11,887 for hemiarthroplasty (p = 0.01), where the cost of the length of stay constituted about 75% of the total (Table 3). The average length of stay in the hemiarthroplasty group was 10.2 days (SD 12) and it was 8.2 days (SD 7.4) in the internal fixation group (p = 0.1). The minimum stay in both groups was 1 day, and the maximum stay was 109 days in the hemiarthroplasty group and 46 days in the internal fixation group. When including the costs of the reoperations and formal and informal outpatient contacts, the total hospital and rehabilitation costs due to the femoral neck fracture were €21,709 for internal fixation and €19,976 for hemiarthroplasty (p = 0.4). 34 patients in the internal fixation group and 28 patients in the hemiarthroplasty group who previously lived at home became permanent nursing home occupants after the fracture, representing a cost of €16,167 per patient in the internal fixation group and €12,281 per patient in the hemiarthroplasty group during follow-up (p = 0.3). The total costs of the interventions when all hospital costs, fracture-related and otherwise, and community-based care was included, were €47,186 for internal fixation and €38,615 for hemiarthroplasty (p = 0.09) (Table 3).

**Table 3.** Costs according to type of cost and intervention. Costs are given in euros (95% CI) and have been adjusted to 2006 figures

Activity (costs)	Hemiarthroplasty	Internal fixation	p-value
**1. Hospital and rehabilitation costs due to the femoral neck fracture**			
a) Primary hospital stay			
i) Running expenses of operation	1,495 (1,455–1,535)	583 (527–640)	< 0.001
ii) Operating personnel	480 (446–514)	267 (240–294)	< 0.001
iii) Inpatient days including postoperative ward	8,827 (6,989–10,665)	7,204 (6,125–8,282)	0.2
iv) Laboratory, medication, X-ray and blood transfusion	363 (322–404)	264 (231–299)	< 0.001
v) Transportation and admission	725 (707–744)	728 (711–746)	0.8
Sum of costs of primary hospital stay (1a)	11,887 (10,126–13,647)	9,044 (7,905–10,189)	0.01
b) Rehabilitation after primary operation	6,415 (4,987–7,843)	4,906 (3,457–6,356)	0.2
Sum of costs of primary hospital stay including rehabilitation (1a + 1b)	18,301 (15,995–20,608)	13,951 (12,017–15,885)	< 0.004
c) Subsequent hospital stay(s) due to reoperations, including rehabilitation	1,644 (521–2,767)	7,684 (5,251–10,117)	< 0.001
d) Further patient contacts during follow-up	30 (14–47)	74 (48–100)	0.01
Sum of hospital costs for fracture treatment (1 a–d)	19,976 (17,478–22,473)	21,709 (18,373–25,044)	0.4
**2. Hospital costs (other than for femoral neck fracture)**			
Hospital costs (other than femoral neck fracture)	2,273 (1,370–3,177)	2,112 (1,309–2,915)	0.8
**3. Costs due to changes in living situation**			
a) Changes in living situation	12,281 (7,017–17,546)	16,167 (10,291–22,043)	0.3
b) Home nursing	3,650 (1,681–5,619)	6,786 (3,742–9,829)	0.09
c) Physiotherapy	433 (248–617)	410 (267–552)	0.8
Sum of costs due to changes in living situation (3 a–c)	16,365 (10,735–21,996)	23,364 (17,051–29,677)	0.1
**Total costs (1–3)**	38,615 (32,362–44,868)	47,186 (39,892–54,479)	0.09

### Revision surgery

In the internal fixation group 47 patients (42%) underwent 70 reoperations, and in the hemiarthroplasty group 11 patients (10%) underwent 13 reoperations (< 0.001) When all patients were included, both reoperated and not reoperated, the distributed extra cost of reoperations for the whole group was €7,684 per patient in the internal fixation group and €1,644 per patient in the hemiarthroplasty group (< 0.001) (Table 3). Using ANOVA with reoperated internal fixation as the reference group, the mean expected additional cost per patient who underwent revision surgery was €33,087 for internal fixation and €20,334 for hemiarthroplasty (p = 0.4). Summing up all the costs in a two-year perspective, the cost of reoperated internal fixation was €66,388 and the cost of reoperated hemiarthroplasty was €56,916 (p = 0.49). The mean length of stay per patient for the reoperations was 0.8 days (SD 3.4) for the hemiarthroplasty group and it was 4.5 days (SD 8.9) for the internal fixation group (< 0.001).

### Sensitivity analysis

The fact that the average cost per patient was similar in the two intervention groups was partly due to the rate of reoperations. Even if there had been no reoperations in the internal fixation group, the total costs would not have been equal between the groups (Table 3). Also, neither halving nor doubling the cost per inpatient day changed the relationship between costs, or the overall conclusions—except that when doubling the cost per day, there was no longer a statistically significant difference between the treatments after the first stay (1a in Table 3).

## Discussion

A common belief has been that hemiarthroplasty is too expensive a treatment for the kinds of patients recruited for this study. The primary treatment was indeed cheaper for the patients in the internal fixation group, mainly due to the shorter hospital stay, but also because of a cheaper operation. When re-admissions due to complications were included, however, hemiarthroplasty was cheaper because of the much higher risk of a reoperation after internal fixation. The difference in the cost due to length of stay after the first admission was more than outweighed by the greater number of inpatient days due to reoperations in the internal fixation group. When other hospital costs and the costs of home-based care were included, the tendency for hemiarthroplasty to be the least expensive treatment was strengthened, although this was still not statistically significant (Table 3).

Our main findings agree with the results of previous studies comparing the cost of treatment after femoral neck fracture. Comparing internal fixation with total hip arthroplasty, Johansson et al. (2006) found a cost of €13,000 in hospital costs for both groups over 2 years. Iorio et al. (2001) estimated hospital costs in the internal fixation group to be $25,000 and in the bipolar hemiarthroplasty group to be $22,000 (US dollars). Rogmark et al. (2003) found a 2-year cost of $21,000 in the internal fixation group and $15,000 in the arthroplasty group, including hospital, rehabilitation, and nursing home costs. Keating et al. (2006) found that internal fixation was more costly than both hemiarthroplasty and total hip arthroplasty. Both Rogmark and Keating found the same pattern as we did, with the initial inpatient episode being less costly in the internal fixation group, but that the subsequent stays more than made up for this in the analysis of total costs.

Our analysis has some weaknesses. Firstly, patients with femoral neck fracture have a substantial mortality. A potential problem with a direct cost analysis is that having died may be perceived as positive, as it does not generate costs. In this study, 35% of patients in each group died during the follow-up period. The difference in total cost was therefore not connected to the mortality. The completeness of cost data can always be questioned. The use of general practitioners or ambulatory specialists outside the hospital was not registered; nor was use of informal help, e.g. from relatives or neighbors. This may be a source of underestimation of the costs associated with patients living in their own home—especially for the internal fixation group, as they had more complications (and therefore more reoperations) and might need more help. Another weakness is the lack of connection between the cause and effect. Was the hip fracture the cause of the increased need for help at home or the reason that some patients became permanent occupants of nursing homes? A hospital admission for pneumonia or a urinary tract infection may or may not be related to the fracture and its treatment. This has no influence on the comparison between groups, but may affect the answer to the question of how much a femoral neck fracture costs. A third possible weakness is the lack of accuracy in the valuations. Compromises were made in finding the exact price of each item and in finding the exact cost consumption per patient or occurrence.

An advantage of our cost study is that it was detailed, thorough, and complete. Furthermore, all patients with femoral neck fractures—including the oldest and the weakest—and patients with cognitive failure were recruited to the study. This gives the study a high degree of generalizability. We chose to maintain a high level of complexity in the analysis, in addition to including as many cost items as possible, both directly and indirectly related to the fracture and its treatment. Our results will be valid for this patient group as a whole and will thus be useful in a decision-making process. The length of stay in hospital was the most important hospital cost in both treatment groups. Other important costs were those of inpatient rehabilitation after the index surgery. The greatest cost, however, was due to nursing home occupancy for some patients. For the internal fixation group the cost of reoperations, further hospital stays, and rehabilitation represented a substantial cost. Reduction of the reoperation rate might be an area with possible savings, but the sensitivity analysis showed that the reoperation rate in the internal fixation group even when reduced to an unrealistically low number, did not equal the costs in the hemiarthroplasty group.

Our study highlights some topics for further research. A cost utility analysis where the cost is viewed in relation to health benefits, such as the cost per quality-adjusted life-year (QALY) or life-years gained, would be of interest. Furthermore, it might be worthwhile to improve the cost analysis in itself by exploring the need for formal and informal help, the help from friends and family, and to get a better registration of the circumstances in which the femoral neck fracture is fully, or in part, to blame for the consumption of resources. An advantage of a pure cost analysis is that all patients may be included, not only the ones for whom information on outcome, such as QALYs, has been possible to gather.
